# Circulating Fibroblast Growth Factor-23 Levels Can Predict Rapid Kidney Function Decline in a Healthy Population: A Community-Based Study

**DOI:** 10.3390/biom13010031

**Published:** 2022-12-24

**Authors:** Hsing-Yu Chen, Wei-Ching Fang, Shao-Chi Chu, Po-Hsi Wang, Chin-Chan Lee, I-Wen Wu, Chiao-Yin Sun, Heng-Jung Hsu, Chun-Yu Chen, Yung-Chang Chen, Vin-Cent Wu, Heng-Chih Pan

**Affiliations:** 1 School of Traditional Chinese Medicine, College of Medicine, Chang Gung University, Taoyuan 33302, Taiwan; 2 Graduate Institute of Clinical Medical Sciences, College of Medicine, Chang Gung University, Taoyuan 33302, Taiwan; 3 Division of Chinese Internal Medicine, Center for Traditional Chinese Medicine, Chang Gung Memorial Hospital, Taoyuan 333, Taiwan; 4 Department of Family Medicine, Linkou Chang Gung Memorial Hospital, Taoyuan 333, Taiwan; 5 Division of Nephrology, Department of Internal Medicine, Keelung Chang Gung Memorial Hospital, Keelung 204, Taiwan; 6 College of Medicine, Chang Gung University, Taoyuan 33302, Taiwan; 7 Community Medicine Research Center, Keelung Chang Gung Memorial Hospital, Keelung 204, Taiwan; 8 Division of Nephrology, Department of Internal Medicine, Linkou Chang Gung Memorial Hospital, Taoyuan 333, Taiwan; 9 Division of Nephrology, Department of Internal Medicine, National Taiwan University Hospital, Taipei 100, Taiwan; 10 Graduate Institute of Clinical Medicine, College of Medicine, National Taiwan University, Taipei 100, Taiwan

**Keywords:** biomarker, chronic kidney disease, FGF-23, rapid kidney function decline

## Abstract

Background: Fibroblast growth factor-23 (FGF-23) associates with decreased kidney function in patients with chronic kidney disease (CKD). However, the correlation between circulating FGF-23 levels and the rate of renal function decline in healthy individuals is largely unknown. We aimed to evaluate the predictive performance of FGF-23 for rapid kidney function decline (RKFD) in a community-based study. Methods: A total of 2963 people residing in northern Taiwan were enrolled from August 2013 to May 2018 for an annual assessment of kidney function for five years. The baseline estimated glomerular filtration rates (eGFR) were calculated using the 2009 and 2021 Chronic Kidney Disease Epidemiology Collaboration (CKD-EPI) equation, which aggregates the values of serum creatinine and cystatin C (eGFRcr-cys). The outcome was RKFD—a 15% decrease in estimated glomerular filtration rate (eGFR) within the first four years, and a reduction in eGFR without improvement in the 5th year. A generalized additive model (GAM) was used to determine the cut-off value of FGF-23 to predict RKFD. Results: The incidence of RKFD was 18.0% (114/634). After matching for age and sex at a 1:1 ratio, a total of 220 subjects were analyzed. eGFRcr-cys was negatively correlated with total vitamin D level but seemed irrelevant to FGF-23. Multivariable logistic regression analysis showed that FGF-23, eGFRcr-cys, and urine albumin-to-creatinine ratio (UACR) were independent predictors of the possibility of RKFD. FGF-23 showed the best predictive performance for RKFD (AUROC: 0.803), followed by baseline eGFRcr-cys (AUROC: 0.639) and UACR (AUROC: 0.591). From the GAM, 32 pg/mL was the most appropriate cut-off value of FGF-23 with which to predict RKFD. The subgroup and sensitivity analyses showed consistent results that high-FGF-23 subjects had higher risks of RKFD. Conclusions: Circulating FGF-23 level could be a helpful predictor for RKFD in this community-based population.

## 1. Background

Chronic kidney disease (CKD) is a long-term condition involving gradual kidney function loss. It has been recognized as a global public health problem due to an increasingly aged population and associated co-morbidities [1]. In addition, CKD patients often have considerable morbidity and mortality due to cardiovascular complications [2,3]. Given the high medical expenses resulting from CKD-related complications [3], identifying subjects at risk for rapid kidney function decline (RKFD) and enacting timely interventions to avoid the following CKD are significant public health concerns [4,5]. RKFD has been found to be associated with higher mortality in older adults and a higher incidence of cardiovascular diseases [6,7,8]. Furthermore, the pathogenesis of subsequent CKD is complex and multifactorial, and disturbances in mineral metabolism are associated with the occurrence of CKD [9,10,11,12,13]. However, the roles of mineral metabolism markers in renal function decline in healthy individuals are largely unknown [5].

Fibroblast growth factor-23 (FGF-23) is a protein hormone mainly synthesized and secreted by osteoblasts and osteocytes [14,15]. It is an essential regulator of mineral ion homeostasis and bone mineralization [16,17,18]. In CKD, FGF-23 is the earliest detectable marker of mineral metabolism, and circulating FGF-23 levels increase before changes in serum levels of phosphate, calcium, or parathyroid hormone [19]. FGF-23 has emerged as an early biomarker of cardiovascular change [20], and circulating FGF-23 connects to the primary adverse clinical outcomes in CKD, such as ESRD, cardiovascular events, and mortality [21]. Furthermore, increased expression of FGF-23 has been associated with the decreased renal function of patients with early CKD [22,23]. A recent study also found that high FGF-23 levels are associated with the risk of new-onset CKD in the general population [24]. The primary physiological role of FGF-23 is to regulate the excretion of urinary phosphate to maintain mineral ion homeostasis in healthy subjects. However, in individuals with good renal function, circulating FGF-23 levels were not associated with serum phosphate levels. To the best of our knowledge, the evidence of a correlation between circulating FGF-23 levels and the rate of renal function decline in healthy individuals is largely unclear.

Recently, the 2021 Chronic Kidney Disease Epidemiology Collaboration (CKD-EPI) proposed the creatinine–cystatin C equation [25], which appears to be less influenced by ethnicity or muscle mass [26] and may achieve greater accuracy and reduce clinical bias in estimating renal function [27,28]. Nevertheless, the clinical usefulness of the 2021 CKD-EPI creatinine–cystatin C equation is still under evaluation. For example, a previous study reported that this newly developed equation yielded higher eGFR than the traditional 2009 CKD-EPI equation in the general European population [29]. Since eGFR is a vital kidney parameter frequently used in clinical practice, the potential clinical significance of the eGFR calculated by the 2021 CKD-EPI creatinine–cystatin C equation (eGFRcr-cys) should be assessed by more different populations. Furthermore, considering that eGFRcr-cys may more accurately reflect renal function, it is questionable whether circulating FGF-23 levels have additional prognostic value compared to eGFRcr-cys alone for RKFD in healthy individuals.

Identifying potential predictors of RKFD may enable risk surveillance and targeted primary prevention of CKD development. Therefore, we hypothesized that the circulating FGF-23 level could provide additional predictive value of detecting subjects at risk of RKFD along with eGFRcr-cys in the healthy population. Thus, the present study aimed to explore the association between circulating FGF-23 and the occurrence of RKFD. Moreover, the predictive performances of FGF-23 and eGFRcr-cys for RKFD were also assessed in this study.

## 2. Materials and Methods

### 2.1. Ethics Statement

This study was conducted in compliance with the ethical principles stated in the Declaration of Helsinki and with the Good Clinical Practice guidelines and local regulatory requirements. The subjects were invited to participate in this study on the day of a health screening visit. Trained nurses evaluated the mental status of all participants during the screening and then explained the informed consent procedures before collecting subjects’ data. This study was approved by the Institutional Review Board of Chang Gung Memorial Hospital (approval no. 201800277B0C601).

### 2.2. Patient Information and Data Collection

This longitudinal, community-based study was conducted in Northeastern Taiwan’s four districts: Wanli, Anle, Ruifang, and Gongliao. The remote locations of these four districts homogenized the medical utilization for the residents and therefore made the community-based study feasible [5]. The community outreach health screening program was performed to recruit subjects, including physical examinations, blood and urine laboratory tests, and a questionnaire survey. During the recruitment period, 2608 individuals recruited from August 2013 to January 2015 completed the baseline survey and joined in the 5-year annual follow-up. Of those invited, 638 attended and 1969 declined. A standardized questionnaire was provided to all the participants by a well-trained team of interviewers to obtain each participant’s information, including drinking, smoking, and betel nut chewing habits; exercise regime; medication history (oral hypoglycemic agents, insulin injections, statins, herbs, and hormones); family history; and physical and mental health status. All the participants agreed to sign informed consent forms.

Basic physical measurements, including blood pressure (mmHg), body weight (kg), and body height (cm), were measured. The laboratory data included biochemistry, inflammatory, and metabolic syndrome-associated markers. Urine samples were collected to evaluate albuminuria by calculating the urine albumin creatinine ratio (UACR). In addition, systemic disorders, such as hypertension, hyperlipidemia, and CKD, were recorded. The exclusion criteria were patients with the following conditions: (1) eGFR < 30 min/mL/1.73 m^2^, (2) no follow-up, (3) unwillingness to participate in the study period, and (4) having undergone organ transplantation or renal replacement therapy before this study. Follow-up data collection was conducted every year after enrollment, and the physical parameters, blood and urine laboratory tests, and questionnaire surveys were recorded. At the end of the study, 619 subjects had completed five years of annual follow-up, 13 had died, and 6 were lost to follow-up.

### 2.3. Definitions of Covariates 

According to the National Kidney Foundation K/DOQI classification, CKD was defined as persistent proteinuria or an eGFR of <60 mL/min/1.73 m^2^, determined using the abbreviated Modification of Diet in Renal Disease equation [30]. Metabolic syndrome was defined as the presence of three out of five of the following criteria according to the National Cholesterol Education Program (NCEP) Adult Treatment Panel III (ATP III) Guidelines [31]: (1) A waist circumference of ≥90 cm in men and ≥80 cm in women according to the modified Asian criteria. (2) Triglycerides ≥ 150 mg/dL or treatment for elevated triglycerides. (3) High-density lipoprotein cholesterol < 40 mg/dL in men or <50 mg/dL in females, or treatment for low high-density lipoprotein cholesterol. (4) Blood pressure ≥ 130/85 mmHg or treatment for hypertension. (5) Fasting glucose ≥ 100 mg/dL or previously diagnosed type 2 diabetes. The HOMA-IR was used to quantify insulin resistance and was calculated as fasting insulin (μIU/mL) × fasting glucose (mg/dL)/405. An increased HOMA-IR score indicates decreased insulin sensitivity [32,33]. BMI was calculated as the body weight divided by the square of the height (kg/m^2^). Meanwhile, several serum mineral biomarkers were measured as the potential candidates for predicting RKFD. The concentrations of serum FGF-23 were determined using a C-terminal enzyme-linked immunosorbent assay kit (R&D Systems, Minneapolis, MN, USA) with 6.1% intra- assay coefficients of variation [34], and the FGF-23 meant the circulating FGF-23 in this study. The serum level of 25 (OH) D was measured using an electro-chemiluminescence immunoassay (Cobas^®^ Vitamin D3 assay, Roche Diagnostics GmbH, Mannheim, Germany) with an inter-assay coefficient of variation of 2.2–13.6% [30]. Each biomarker assay was duplicated according to the manufacturer’s instructions, and the mean value was used for further statistical analysis. 

### 2.4. Outcome Assessment

All eligible participants were followed up for five years from the index date. The primary outcome of this study was RKFD, which has been defined as a decline in eGFR of ≥30% within ten years or an absolute annual loss ≥ 3 mL/min/1.73 m^2^ [7]. In this 5-year longitudinal follow-up study, we modified RKFD’s definition as a 15% decline in eGFR within the first four years and a reduction in eGFR which did not improve in the 5th year [5]. 

### 2.5. Statistical Analysis

Continuous variables are summarized as medians and interquartile ranges (the distance between the first and third quartile) or means and standard deviations, depending on the nature of the data distribution. The student’s t-test was used to compare the means of continuous variables and normally distributed data; otherwise, the Mann–Whitney U test was used. Categorical data were tested using the *χ*^2^-statistic. Correlations of paired-group variables were assessed using Pearson analysis. Discrimination was evaluated using the area under the receiver operating characteristic curve (AUROC) values, and a value close to 0.5 indicates that the model’s performance approximates that of flipping a coin. The AUROC values were compared using a nonparametric approach. Furthermore, risk factors and candidate predictors were assessed with univariate and multivariable logistic regression analyses, in which only significant covariates were further evaluated as the final predictors of RKFD. A generalized additive model (GAM) was plotted and adjusted for co-morbidities, sex, and age in individual patients, in which the non-linear nature of relations between independent and dependent covariates was the rationale to use GAM in this study [35,36]. The model incorporated subject-specific random effects, expressed as the logarithm of the odd (logit), and the optimal cut-off value was defined as a log odds value of zero [37]. Using GAM to obtain the cut-off value of FGF-23 would be closer to the real-world situation than using a single predictor alone, since the GAM could consider accessible covariates simultaneously [36]. Cumulative survival curves as a function of time were plotted using the Kaplan–Meier method and compared with the log-rank test. Additionally, the Cox regression with and without considering the assessable covariates was used to estimate the risk of the 5-year occurrence of RKFD.

Furthermore, subgroup analyses for RKFD were performed, including age (>60 and ≤60 years), sex, and co-morbidities (hypertension, diabetes, metabolic syndrome, CVD, and gout); and interactions between FGF-23 and the covariates were also examined. To validate the study results, sensitivity tests with different propensity score (PS) models were performed [38]. For different PS models, inverse probability of treatment weighting (IPTW), 1:1 propensity score matching (PSM), and 5-block stratification were used. All statistical tests were two-tailed. A value of *p*-value < 0.05 was considered statistically significant. Furthermore, we used the decision curve analysis (DCA) to evaluate the benefit of using FGF-23 to predict the occurrence of RKFD, and we also calculated the net reclassification improvement (NRI) and integrated discrimination improvement (IDI) to estimate the overall improvement in reclassification with FGF-23 in contrast to eGFRcr-cys based on demographic features [39,40,41,42,43]. Data were analyzed using SPSS version 22.0 software (SPSS, Inc., Chicago, IL, USA) and Stata (StataCorp. 2019. Stata Statistical Software: Release 16. College Station, TX, USA: StataCorp LLC.).

## 3. Results

### 3.1. Characteristics of the Study Subjects

**Figure 1:**
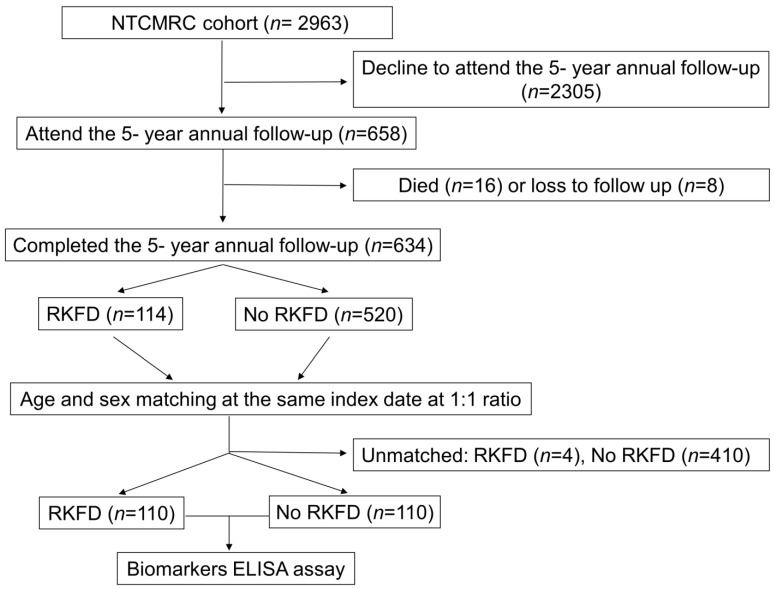
Flow diagram of this study. First case: 20130810, last case 20150124. Abbreviations: ELISA, enzyme-linked immunosorbent assay; NTCMRC, Northeastern Taiwan Community Medicine Research Cohort; RKFD, rapid kidney function decline.

Among the 634 subjects, 114 (18.0%) developed RKFD during the 5-year study period. To determine the correlations between the biomarkers (FGF-23, total vitamin D, UACR, and eGFRcr-cys) and RKFD, we further matched the subjects by age and sex at the same index date at a 1:1 ratio. The study’s flowchart is shown in Figure 1. A total of 220 subjects were included for further analysis: 110 in the group with RKFD and 110 in the group without RKFD. Four individuals were excluded after 1:1 matching of age and sex due to female predominance and older age.

The baseline characteristics of the two groups are shown in Table 1. The mean age of the subjects was 58.8 years, and 46 subjects were men (20.9%). The RKFD group had a higher prevalence of metabolic syndrome and central obesity; higher levels of eGFR, eGFRcr-cys, urine albumin-creatinine ratio (UACR), fasting glucose, insulin, HOMA-IR, and FGF-23; and lower levels of total cholesterol, LDL, and serum creatinine than the group without RKFD. In terms of medication, the use of both oral hypoglycemic agents and analgesics was higher in the RKFD group. We also compared the social psychology variables of the study population (Supplemental Table S1). The two groups had similar education levels, substance use, and diet habits.

### 3.2. Analysis of Factors Associated with the Possibility of RKFD

**Figure 2:**
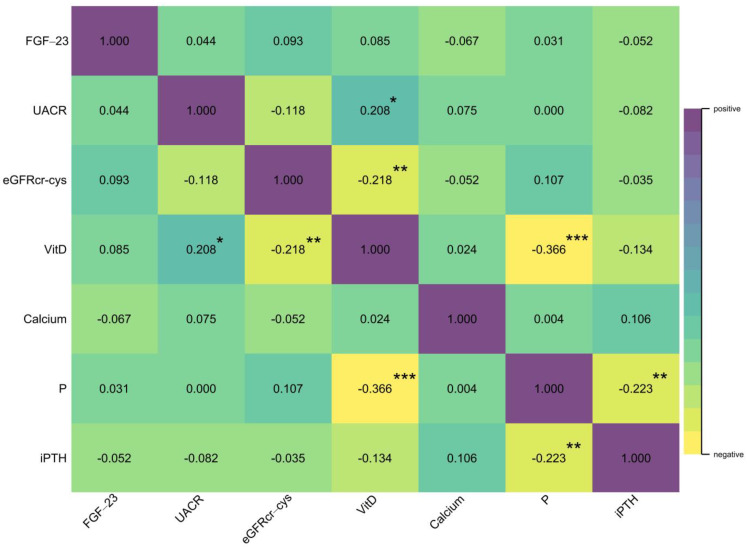

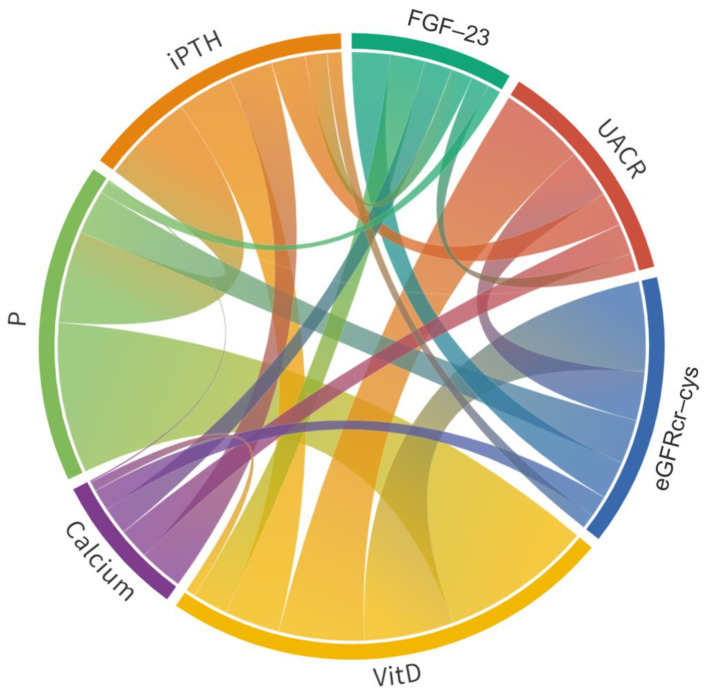
Correlations among FGF-23, UACR, eGFRcr-cys, vitamin D, calcium, inorganic phosphorus, and intact PTH. (* *p*-value < 0.05; ** *p*-value < 0.01; *** *p*-value < 0.001).

We examined the correlations among eGFRcr-cys, UACR, and biomarkers serum levels at the study’s beginning (Figure 2). eGFRcr-cys was significantly negatively correlated with the level of total vitamin D, and total vitamin D was negatively correlated with the level of serum inorganic P but not serum calcium or intact PTH. Moreover, FGF-23 seemed relatively independent of UACR, eGFRcr-cys, and total vitamin D.

**Figure 3:**
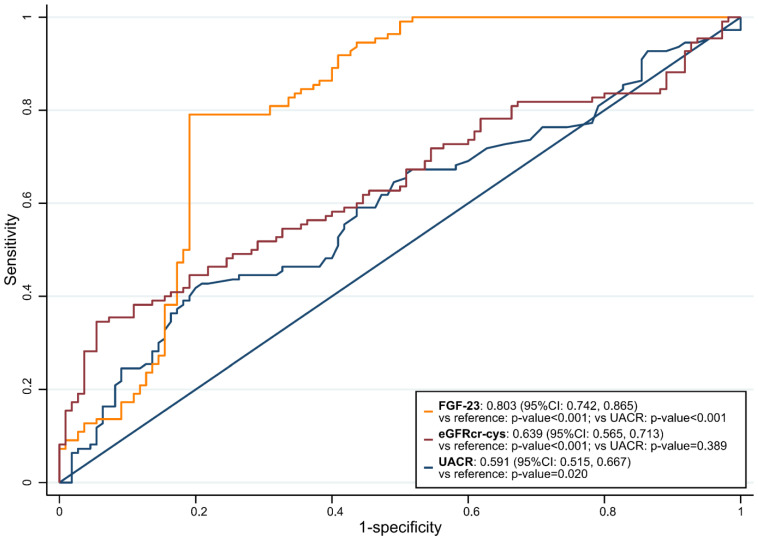
The areas under the receiver operating characteristic (AUROC) curve for biomarkers to predict the occurrence of 5-year RKFD.

The univariate regression shows 11 of the 44 variables (Table 1) were feasible prognostic indicators. On performing multivariable regression analysis, we identified that the baseline FGF-23 (adjusted odds ratio (aOR): 2.87, 95% confidence interval (CI): 1.47, 5.62), eGFRcr-cys (aOR: 2.50, 95% CI: 1.54, 4.05), urine albumin-creatinine ratio (UACR) (aOR: 1.16, 95% CI: 1.03, 1.32), and central obesity (aOR: 5.09, 95% CI: 1.15, 22.60) had independent prognostic significance for the likelihood of RKFD (Table 2). Furthermore, based on AUROC analysis, FGF-23 had significantly better discriminatory power for RKFD than UACR (AUROC: 0.803, 95% CI: 0.742, 0.865 versus AUROC: 0.591, 95% CI: 0.515, 0.667, respectively) and eGFRcr-cys (AUROC: 0.639, 95% CI: 0.565, 0.713) (Figure 3). 

### 3.3. FGF-23 Could Predict RKFD in the Healthy Population

**Figure 4:**
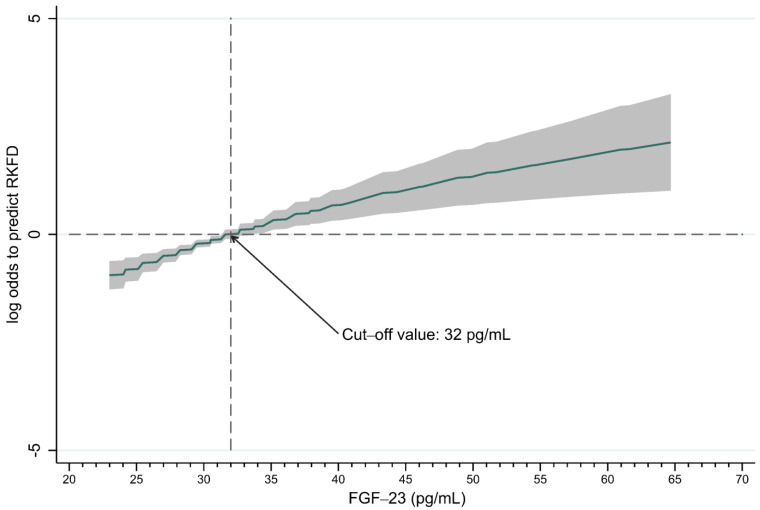
Generalized additive model plot for the FGF-23 (cut-off value: 32 pg/mL) against the probability of RKFD (adjusted with eGFRcr-cys, UACR, age, gender, metabolic syndrome, total cholesterol, LDL).

We used a non-linear GAM to identify adequate cut-off values of FGF-23 to predict RKFD (Figure 4). All the relevant covariates listed in Table 1, including age, gender, metabolic syndrome, lipid profile, UACR, eGFRcr-cys, and FGF-23, were included in GAM. The results show that a high FGF-23 level (cut-off value: 32 pg/mL) was associated with a higher possibility of RKFD. We further divided the patients into high (≥32 pg/mL) and low (<32 pg/mL) FGF-23 level groups (Table 3). The incidence of RKFD in the high-FGF-23 group was significantly higher than that in the low-FGF-23 group (70.2% in the high-FGF-23 group vs. 42.9% in the low-FGF-23 group, *p*-value < 0.001). In addition, the high-FGF-23 group was significantly younger (55.9 versus 59.8 years, *p*-value = 0.020) and had 9 mL/min/1.73 m^2^ higher levels of eGFRcr-cys (*p*-value = 0.009). On the other hand, the high-FGF-23 group had 5 mmHg lower systolic blood pressure on average (*p*-value = 0.041), had a lower average body mass index by 1 kg/m^2^ (*p*-value = 0.039), and about 15% less central obesity (*p*-value = 0.04).

**Figure 5:**
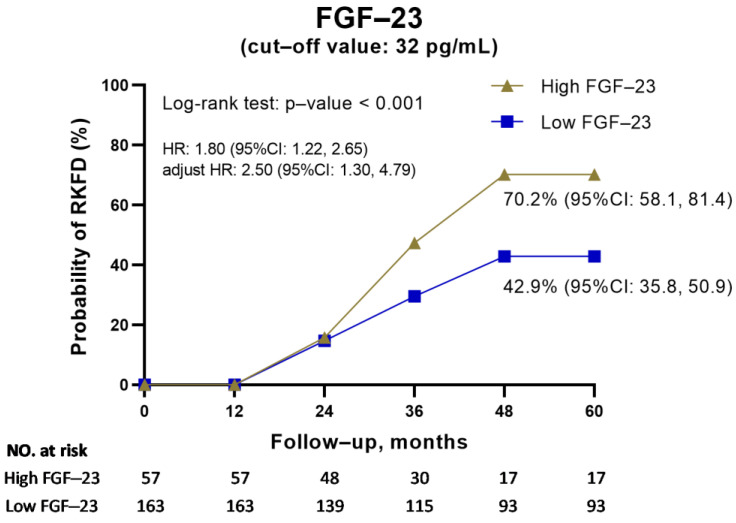
Kaplan–Meier estimation of the probability of the occurrence of RKFD stratified by FGF-23.

Figure 5 illustrates stratified cumulative probabilities of the occurrence of RFKD according to FGF-23 level and demonstrates that the high-FGF-23 group had a significantly higher cumulative RKFD rate than the low-FGF-23 group (high vs. low-FGF-23 group = 70.2% vs. 42.9%, log-rank test *p*-value < 0.001). Moreover, when considering time factors, the Cox regression showed the high-FGF-23 subjects were significantly associated with a higher risk of the occurrence of RKFD than low-FGF-23 subjects (hazard ratio (HR): 1.80, 95% CI: 1.22, 2.65; adjusted hazard ratio (aHR): 2.50, 95% CI: 1.30, 4.79; Figure 5 and Supplemental Table S2).

Furthermore, NRI and IDI analyses showed a similar positive trend of using FGF-23 to predict RKFD, and the DCA showed the benefit of using FGF-23 for all probabilities of RKFD (Supplemental Figure S1; Supplemental Table S3). Incorporating FGF-23 with base covariates led to a significant increase in risk stratification (continuous NRI = 0.384; standard error: 0.172; *p*-value = 0.026). Most of this effect came from the subjects without RKFD (event IDI = 0.073; standard error: 0.017; *p*-value < 0.0001). Of note, the subjects with a high FGF-23 level had a significantly higher eGFR on average than those with a low FGF-23 level at all time points. However, they had a more significant reduction in eGFR during the study period (high vs. low-FGF-23 group = 18.73% vs. 14.77%, *p*-value < 0.001) (Supplemental Figure S2). 

### 3.4. Subgroup and Sensitivity Analyses

Supplemental Table S4 shows the results of the subgroup analysis. The trends of the risk for RKFD were similar among all subpopulations, and interactions between FGF-23 ≥ 32 pg/mL and stratified covariates were found. The association between high FGF-23 levels and a higher risk of RKFD was more significant in patients with younger age and cardiovascular disease and those without hypertension, metabolic syndrome, or gout. Supplemental Table S5 shows the consistent associations between high-FGF-23 patients and higher risks of RKFD based on different PS-based models.

## 4. Discussion

We analyzed 220 healthy subjects residing in northern Taiwan to study the association between circulating level of FGF-23 and the risk of RKFD. At the same time, the clinical significance of eGFRcr-cys was also assessed. The main findings were as follows: First, the incidence of RKFD was 18.0% (114/634), which agrees with the previous study [5]. Second, the serum FGF-23 level could be used with UACR and eGFRcr-cys as an approximation of kidney function to forecast RKFD among community subjects. Among these predictors, FGF-23 seemed the most valuable predictor. Third, our results demonstrated that a high level of FGF-23 (≥ 32 pg/mL) was associated with a higher risk of developing RKFD in this healthy population, which provides a crucial reference for clinical practice. By using the Cox regression, subjects with FGF-23 ≥ 32 pg/mL had a 2.5 times higher risk of the occurrence of RKFD than subjects with low FGF-23 when considering covariates about demographic features, including metabolic profiles and mineral homeostasis. These results showed the potential role and reference value of FGF-23 in predicting RKFD independently. Finally, the association between a high FGF-23 level and a high risk of RKFD was consistent across subgroups of age, sex, CVD, hypertension, diabetes, metabolic syndrome, and gout. These results remained robust in the sensitivity analyses. 

The roles of FGF-23 and total vitamin D as kidney function declines in patients with CKD have been reported in previous studies [22,23,44]. Furthermore, we found that baseline FGF-23 had strong discriminatory power for RKFD. However, the mechanisms of RKFD are complex and multifactorial. FGF-23 and total vitamin D help maintain the homeostasis between calcium and phosphate levels as kidney function declines and maintain mineral homeostasis in early CKD [45]. Moreover, several studies reported the vital role of FGF-23 in inflammation regulation and abnormal metabolism of renal vitamin D and phosphate in the early CKD animal model [46,47]. Renal impairment disturbs the balance of calcium and phosphate metabolism. It may directly lead to cardiovascular calcification and systemic inflammation, which may be why the level of FGF-23 is associated with RKFD [46,48]. In our study, FGF-23 was irrelevant to the serum levels of vitamin D, inorganic P, and intact PTH; and only FGF-23 had strong associations with the occurrence of RKFD in five years. We also found that the association between a high FGF-23 level and RKFD was more significant in the subjects with cardiovascular disease. All the above suggests that an increase in FGF-23 may indicate an early disturbance of mineral homeostasis and thus become reasonable in stratifying the risk of RKFD subjects. 

Additionally, FGF-23 had additional predictability when combined with eGFR for RKFD in healthy people. In the literature, UACR is an essential indicator for evaluating the severity of CKD and a good predictor for renal progression in diabetic patients [49,50]. Our model suggests that the levels of UACR were independently associated with RKFD risk in healthy people. However, the discriminatory power of UACR for RKFD was not good or significantly better than that of conventional eGFR. A possible explanation is that the prevalence of significant proteinuria (UACR ≥ 30 mg/g) was extremely low in our study subjects, which reduced the predictive performance of UACR. On the other hand, in subgroup analysis, we found that the trend of the association between a high FGF-23 level and RKFD was more significant in the younger subjects and those without hypertension or metabolic syndrome. Given that aging, hypertension, and metabolic syndrome are major causes of CKD [9,12,51], FGF-23 appears to have important clinical implications for assessing the risk of RKFD in healthy subjects. These findings support our hypothesis that incorporating biomarkers into clinical practice may improve clinical decision making in screening subjects at risk of RKFD. Furthermore, the early identification of subjects at risk of RKFD may allow for timely and targeted interventions.

Despite the encouraging results observed in this study, several potential limitations should be recognized. First, some confounders were not measured and thus warranted consideration. For example, the use of only time point measurement of FGF-23 instead of sequential FGF-23 measurement provided only cross-sectional data. Additionally, the fact that our study involved patients of the same ethnicity limits the generalizability of the findings to other regions with different ethnic populations. Second, the predictive value of FGF-23 for the risk of mortality has been well documented in many clinical scenarios [52,53]. However, serum FGF-23 level was not measured sequentially in this study, and sequential measurements of serum FGF-23 with the highest and lowest values may be more useful predictive markers than initial serum FGF-23 level alone. However, the timing of the occurrence of the highest and lowest serum FGF-23 level is not specific, so it is not easy to apply clinically. Third, most of the participants were female (77.5%), and thus, the generalizability may be limited. Finally, we also acknowledge that the observational nature of the study without a pre-specified protocol for the intervention cannot conclude on causal relationships. Therefore, we can only speculate that serum FGF-23 level may be a predictive variable, and further prospective observational studies are needed to validate our results. 

## 5. Conclusions

There is a high incidence of RKFD (more than 15%) in the communities we assed. Our results showed that a serum FGF-23 level ≥ 32 pg/mL could be considered an independent risk factor for RKFD. We suggest that serum FGF-23 level is accurate and capable of assessing the risk of RKFD in healthy subjects. These results provide important implications for using FGF-23 in predicting the RKFD, which may alert doctors and patients to the subsequent risk of CKD.

## Figures and Tables

**Table 1:** Baseline characteristics of the study population.

	Total (*n* = 220)	RKFD (*n* = 110)	No RKFD (*n* = 110)	*p*-Value
Demographics				
Age, years	58.8 (10.8)	59.3 (10.8)	58.3 (10.9)	0.52
Male gender, n	46 (20.9%)	22 (20.0%)	24 (21.8%)	0.74
Hypertension, n	56 (25.5%)	34 (30.9%)	22 (20.0%)	0.063
DM, n	25 (11.4%)	17 (15.5%)	8 (7.3%)	0.056
CKD, n	6 (2.7%)	4 (3.6%)	2 (1.8%)	0.41
Cardiovascular disease, n	17 (7.7%)	10 (9.1%)	7 (6.4%)	0.45
CVA, n	3 (1.4%)	3 (2.7%)	0 (0.0%)	0.081
HBV, n	28 (12.7%)	14 (12.7%)	14 (12.7%)	1.00
HCV, n	5 (2.3%)	2 (1.8%)	3 (2.7%)	0.65
Gout, n	7 (3.2%)	5 (4.5%)	2 (1.8%)	0.25
Autoimmune disease, n	2 (0.9%)	2 (1.8%)	0 (0.0%)	0.16
Kidney stone	4 (1.8%)	1 (0.9%)	3 (2.7%)	0.31
Mental disorder	3 (1.4%)	2 (1.8%)	1 (0.9%)	0.56
Endocrine disorder	2 (0.9%)	0 (0.0%)	2 (1.8%)	0.16
Metabolic syndrome	63 (28.6%)	43 (39.1%)	20 (18.2%)	**<0.001**
Biochemical and physiological profiles				
SBP, mmHg	129.50 (118.00–141.00)	129.50 (118.00–143.00)	129.50 (119.00–137.00)	0.89
BMI, kg/m^2^	23.95 (22.22–26.47)	24.23 (22.22–26.25)	23.72 (22.21–26.67)	0.71
BMI > 24, n	108 (49.1%)	61 (55.5%)	47 (42.7%)	0.059
Central obesity, n	95 (43.2%)	56 (50.9%)	39 (35.5%)	**0.021**
Hgb, g/dL	13.70 (12.80–14.60)	13.70 (12.60–14.60)	13.60 (13.00–14.60)	0.93
Total cholesterol, mg/dL	207.50 (189.50–230.00)	199.50 (187.00–223.00)	213.50 (195.00–235.00)	**0.011**
LDL cholesterol, mg/dL	121.40 (105.05–144.70)	117.80 (100.40–138.10)	129.90 (109.90–150.60)	**0.016**
HDL cholesterol, mg/dL	56.55 (47.75–66.95)	54.70 (46.60–64.70)	57.50 (48.90–71.80)	0.084
Triglyceride, mg/dL	92.00 (69.00–132.00)	96.00 (69.00–155.00)	90.00 (69.00–127.00)	0.33
BUN, mg/dL	12.00 (10.00–15.00)	12.00 (10.00–16.00)	12.00 (10.00–15.00)	0.97
Creatinine, mg/dL	0.63 (0.55–0.77)	0.61 (0.54–0.75)	0.67 (0.55–0.78)	**0.049**
Alk-P, mg/dL	64.5 (55.00–79.00)	65.0 (54.00–80.00)	64.0 (56.00–76.00)	0.82
eGFRcr-cys, mL/min/1.73 m^2^	101.89 (82.68–114.96)	108.13 (90.75–121.69)	99.32 (80.85–110.07)	**<0.001**
Uric acid, mg/dL	5.10 (4.30–6.10)	5.10 (4.20–6.10)	5.10 (4.40–6.10)	0.93
Albumin, g/dL	4.69 (0.26)	4.69 (0.27)	4.69 (0.25)	0.84
GPT, U/L	21.00 (17.00–30.00)	22.00 (17.00–31.00)	21.00 (17.00–28.00)	0.27
UACR, mg/g	5.90 (3.85–9.50)	6.50 (4.10–11.20)	5.30 (3.70–8.00)	**0.020**
Fasting glucose, mg/dL	96.00 (91.50–104.00)	97.00 (93.00–108.00)	95.00 (91.00–102.00)	**0.038**
HbA1C, %	5.60 (5.40–5.95)	5.60 (5.40–6.10)	5.60 (5.40–5.90)	0.29
Insulin, μIU/mL	5.85 (4.21–9.20)	6.16 (4.68–9.92)	5.25 (3.89–8.58)	**0.048**
HOMA-IR	1.45 (0.95–2.38)	1.54 (1.18–2.58)	1.31 (0.91–2.02)	**0.013**
FGF-23, pg/mL	29.69 (27.10–32.43)	30.73 (30.42–34.34)	27.15 (23.63–29.04)	**<0.001**
Total Vitamin D, ng/mL	24.54 (19.44–31.70)	25.30 (20.16–32.81)	23.98 (19.10–30.77)	0.31
iPTH, mg/dL	44.10 (34.20–58.60)	43.85 (31.60–53.40)	44.60 (35.60–62.30)	0.12
P, mg/dL	3.88 (0.53)	3.90 (0.48)	3.87 (0.58)	0.62
Ca, mg/dL	9.30 (9.15–9.50)	9.30 (9.20–9.60)	9.30 (9.10–9.50)	0.39
Medication use				
OHA, n	24 (11.1%)	17 (15.9%)	7 (6.4%)	**0.027**
Anti-hypertensives, n	51 (23.5%)	31 (29.0%)	20 (18.2%)	0.061
Pain killer, n	30 (14.4%)	20 (19.6%)	10 (9.4%)	0.037

Abbreviations: BMI, body mass index; SBP, systolic blood pressure; BUN, blood urea nitrogen; CKD, chronic kidney disease; CVA, cerebrovascular accident; DM, diabetes mellitus; eGFRcr-cys, estimated glomerular filtration rate by serum creatinine and cystatin C; FGF-23, fibroblast growth factor-23; GPT, glutamic pyruvic transaminase; HbA1C, glycated hemoglobin; HOMA-IR, homeostatic model assessment-insulin resistance; HDL, high-density lipoprotein; LDL, low-density lipoprotein; OHA, oral hypoglycemic agents; RKFD, rapid kidney function decline; UACR, urine albumin-to-creatinine ratio. Values in bold are statistically significant (*p* < 0.05). All numerical covariates are presented as median with 25th–75th percentiles except age, albumin, and P. These three covariates passed the Shapiro–Wilk W test, and therefore, the values of mean with standard deviation were used to describe these three covariates.

**Table 2:** The prognostic significance of our variables for RKFD.

Variables	Beta Coefficient	Standard Error	Odds Ratios (95% CI)	*p*-Value
**Univariable analysis**				
Age, per 10 years	0.08	0.13	1.08 (0.85, 1.39)	0.517
Male	−0.11	0.33	0.90 (0.47, 1.72)	0.740
Hypertension	0.58	0.32	1.79 (0.96, 3.32)	0.065
DM	0.85	0.45	2.33 (0.96, 5.65)	0.061
CKD	0.71	0.88	2.04 (0.37, 11.36)	0.417
Cardiovascular disease	0.39	0.51	1.47 (0.54, 4.02)	0.451
Gout	0.94	0.85	2.57 (0.49, 13.55)	0.265
Metabolic syndrome	1.06	0.32	2.89 (1.56, 5.36)	0.001
BMI, per 1 kg/m^2^	0.51	0.27	1.67 (0.98, 2.84)	0.060
Central obesity	0.64	0.28	1.89 (1.10, 3.24)	0.021
Hgb, per 1 g/dL	−0.02	0.10	0.98 (0.81, 1.19)	0.830
Total cholesterol, per 10 mg/dL	−0.09	0.04	0.92 (0.85, 0.99)	0.029
LDL cholesterol, per 10 mg/dL	−0.09	0.05	0.91 (0.83, 1.00)	0.043
HDL cholesterol, per 10 mg/dL	−0.17	0.09	0.85 (0.71, 1.02)	0.074
Triglyceride, per 10 mg/dL	0.03	0.02	1.03 (0.99, 1.07)	0.175
BUN, per 1 mg/dL	−0.00	0.03	1.00 (0.93, 1.07)	0.890
Creatinine, per 1 mg/dL	−1.59	0.90	0.20 (0.04, 1.19)	0.077
eGFRcr-cys, per 10 mL/min/1.73 m^2^	0.24	0.07	1.27 (1.11, 1.45)	<0.001
Uric acid, per 1 mg/dL	0.03	0.11	1.03 (0.83, 1.28)	0.804
Albumin, per 1 g/dL	0.11	0.52	1.11 (0.40, 3.06)	0.836
GPT, per 10 U/L	0.03	0.06	1.03 (0.92, 1.16)	0.613
UACR, per 1 mg/g	0.07	0.03	1.07 (1.02, 1.14)	0.012
Fasting glucose, per 10 mg/dL	0.17	0.08	1.19 (1.02, 1.39)	0.025
HbA1C, per 1 %	0.45	0.21	1.56 (1.04, 2.35)	0.030
Insulin, per 10 μIU/mL	0.48	0.27	1.62 (0.95, 2.75)	0.077
HOMA-IR	0.19	0.10	1.21 (0.99, 1.46)	0.056
FGF-23, per 10 pg/mL	0.80	0.22	2.23 (1.43, 3.45)	<0.001
Total Vitamin D, per 1 ng/mL	0.02	0.02	1.02 (0.98, 1.05)	0.338
iPTH, per 1 mg/dL	−0.00	0.01	1.00 (0.99, 1.01)	0.634
P, per 1 mg/dL	0.13	0.26	1.14 (0.69, 1.87)	0.619
Ca, per 1 mg/dL	0.29	0.39	1.34 (0.62, 2.89)	0.460
OHA use	1.01	0.47	2.75 (1.09, 6.94)	0.032
Anti-hypertensives	0.61	0.33	1.84 (0.97, 3.48)	0.063
Pain killer use	0.85	0.42	2.34 (1.04, 5.29)	0.041
Vegetarian	0.02	0.36	1.02 (0.51, 2.06)	0.950
Multivariable analysis *				
Central obesity	1.63	0.76	5.09 (1.15, 22.60)	0.032
eGFRcr-cys, per 10 mL/min/1.73 m^2^	0.92	0.25	2.50 (1.54, 4.05)	<0.001
UACR, per 1 mg/g	0.15	0.06	1.16 (1.03, 1.32)	0.017
FGF-23, per 10 pg/mL	1.06	0.34	2.87 (1.47, 5.62)	0.002

Abbreviations: BMI, body mass index; BUN, blood urea nitrogen; CKD, chronic kidney disease; CVA, cerebrovascular accident; DM, diabetes mellitus; eGFRcr-cys, estimated glomerular filtration rate by serum creatinine and cystatin C; FGF-23, fibroblast growth factor-23; GPT, glutamic pyruvic transaminase; HbA1C, glycated hemoglobin; HOMA-IR, homeostatic model assessment-insulin resistance; HDL, high-density lipoprotein; LDL, low-density lipoprotein; OHA, oral hypoglycemic agents; RKFD, rapid kidney function decline; UACR, urine albumin-to-creatinine ratio. * Covariates used in multivariable analysis: sex, hypertension, DM, CKD, cardiovascular disease, CVA, HBV, HCV, gout, autoimmune disease, metabolic syndrome, BMI, central obesity, Hgb, total cholesterol, LDL cholesterol, HDL cholesterol, triglyceride, BUN, eGFRcr-cys, uric acid, albumin, GPT, UACR, fasting glucose, HbA1C, insulin, HOMA-IR, FGF-23, total vitamin D, iPTH, P, Ca, OHA use, anti-hypertensives, pain killer use, and dietary habits.

**Table 3:** Demographic characteristics of enrolled subjects stratified by cut-off values of FGF-23 and 32 pg/mL.

	High FGF-23 ≥32 pg/mL (*n* = 57)	Low FGF-23 <32 pg/mL (*n* = 163)	*p*-Value
RKFD	40 (70.2%)	70 (42.9%)	**<0.001**
Demographics			
Age, years	55.9 (11.0)	59.8 (10.6)	**0.020**
Male gender, n	15 (26.3%)	31 (19.0%)	0.24
Hypertension, n	11 (19.3%)	45 (27.6%)	0.22
DM, n	7 (12.3%)	18 (11.0%)	0.80
CKD, n	3 (5.3%)	3 (1.8%)	0.17
Cardiovascular disease, n	6 (10.5%)	11 (6.7%)	0.36
CVA, n	1 (1.8%)	2 (1.2%)	0.77
HBV, n	9 (15.8%)	19 (11.7%)	0.42
HCV, n	0 (0.0%)	5 (3.1%)	0.18
Gout, n	4 (7.0%)	3 (1.8%)	0.055
Autoimmune disease, n	1 (1.8%)	1 (0.6%)	0.43
Metabolic syndrome	14 (24.6%)	49 (30.1%)	0.43
SBP, mmHg	126.00 (116.00–136.00)	131.00 (119.00–143.00)	**0.041**
BMI, kg/m^2^	23.12 (21.75–25.44)	24.16 (22.28–26.94)	**0.039**
BMI > 24, n	25 (43.9%)	83 (50.9%)	0.36
Central obesity, n	18 (31.6%)	77 (47.2%)	**0.040**
Hgb, g/dL	13.80 (13.10–14.60)	13.60 (12.70–14.60)	0.41
Total cholesterol, mg/dL	206.00 (187.00–235.00)	208.00 (191.00–230.00)	0.50
LDL cholesterol, mg/dL	117.20 (93.70–144.80)	123.70 (108.40–144.60)	0.15
HDL cholesterol, mg/dL	56.40 (46.70–69.40)	57.00 (48.60–66.90)	0.82
Triglyceride, mg/dL	86.00 (61.00–138.00)	97.00 (71.00–132.00)	0.24
BUN, mg/dL	12.00 (10.00–15.00)	12.00 (10.00–15.00)	0.77
Creatinine, mg/dL	0.61 (0.54–0.73)	0.64 (0.55–0.77)	0.31
eGFR-cys, mL/min/1.73 m^2^	109.81 (95.51–121.47)	100.96 (80.85–113.33)	**0.009**
Uric acid, mg/dL	5.00 (4.30–6.00)	5.10 (4.40–6.20)	0.31
Albumin, g/dL	4.66 (0.28)	4.70 (0.26)	0.39
GPT, U/L	22.00 (17.00–31.00)	21.00 (17.00–29.00)	0.95
UACR, mg/g	5.40 (3.50–8.20)	6.40 (4.10–9.90)	0.074
Fasting glucose, mg/dL	95.00 (90.00–101.00)	97.00 (92.00–104.00)	0.064
HbA1C, %	5.60 (5.30–5.80)	5.70 (5.40–6.00)	0.13
Insulin, μIU/mL	5.82 (3.16–8.43)	5.86 (4.48–9.56)	0.12
HOMA-IR	1.45 (0.76–2.10)	1.45 (1.05–2.39)	0.11
FGF-23, pg/mL	39.70 (34.34–54.48)	27.57 (24.52–30.54)	**<0.001**
Total Vitamin D, ng/mL	24.80 (19.28–30.98)	24.29 (19.46–31.70)	0.66
iPTH, mg/dL	40.10 (32.70–51.30)	45.20 (34.80–60.10)	0.16
P, mg/dL	3.88 (0.55)	3.89 (0.53)	0.89
Ca, mg/dL	9.30 (9.10–9.50)	9.30 (9.20–9.60)	0.74
Medication use			
OHA, n	7 (13.0%)	17 (10.5%)	0.62
Anti-hypertensives, n	9 (16.4%)	42 (25.9%)	0.15
Pain killer, n	9 (16.7%)	21 (13.6%)	0.59

Abbreviations: BMI, body mass index; SBP, systolic blood pressure; BUN, blood urea nitrogen; CKD, chronic kidney disease; CVA, cerebrovascular accident; DM, diabetes mellitus; eGFRcr-cys, estimated glomerular filtration rate by serum creatinine and cystatin C; FGF-23, fibroblast growth factor-23; GPT, glutamic pyruvic transaminase; HbA1C, glycated hemoglobin; HOMA-IR, homeostatic model assessment-insulin resistance; HDL, high-density lipoprotein; LDL, low-density lipoprotein; OHA, oral hypoglycemic agents; RKFD, rapid kidney function decline; UACR, urine albumin-to-creatinine ratio. Values in bold are statistically significant (*p* < 0.05). All numerical covariates are presented as median with 25th–75th percentiles except age, albumin, and P. These three covariates passed the Shapiro–Wilk W test, and therefore, the values of mean with standard deviation were used to describe these three covariates.

## Data Availability

The datasets used and/or analyzed during the current study are available from the corresponding author upon reasonable request.

## Supplementary Materials

The following supporting information can be downloaded at: https://www.mdpi.com/article/10.3390/biom13010031/s1, Figure S1: DCA plot to assess the clinical consequences of screening patients for the risk of RKFD using FGF-23 and eGFRcr-cys; Figure S2: Changes in eGFR according to the serum levels of FGF-23 during the study; Table S1: Social psychology variables of the study population; Table S2. Hazard ratios estimated by the Cox regression; Table S3. NRI and IDI analyses for the role of FGF-23 in stratifying individuals into high or low-risk categories (re-classification); Table S4. Subgroup analysis of RKFD compared with low (<32 pg/mL) and high levels (≥32 pg/mL) of FGF-23; Table S5. Sensitivity analysis for risk estimation of FGF-23 ≥ 32 pg/mL.
