# Supplementary material for: Circulating Fibroblast Growth Factor-23 Levels Can Predict Rapid Kidney Function Decline in a Healthy Population: A Community-Based Study
Source: Biomolecules. 2022 Dec 24;13(1):31. doi: 10.3390/biom13010031 (PMC9856057; doi:10.3390/biom13010031)
Supplement: Supplementary file 1 [file biomolecules-13-00031-s001.zip › biomolecules-2047300-supplementary.pdf]

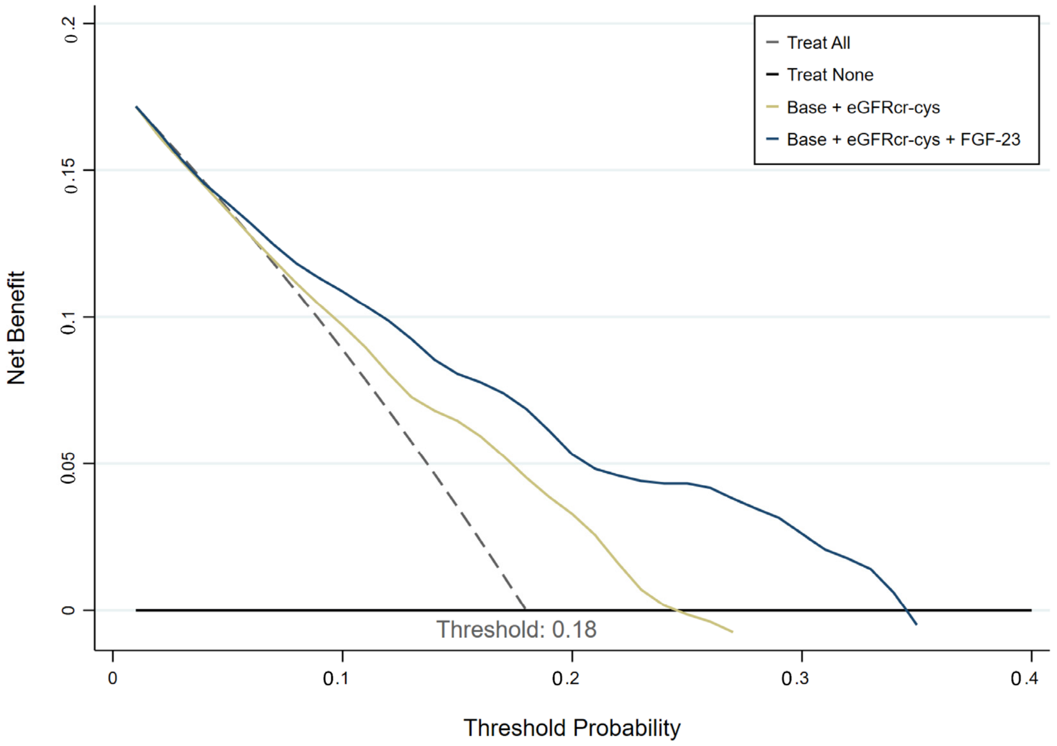

Supplemental Figure S1. DCA plot to assess the clinical consequences of screening patients for the risk of RKFD using FGF-23 and eGFRcr-cys. Y-axis is the net benefit of the decision strategy. Net benefit was defined as the net proportion of subjects with RKFD in whom a prediction model would provide benefit without applying a prediction model to subjects with good outcomes. For the subjects who did not develop RKFD (solid black line), forecasting with a combination of FGF-23 and eGFRcr-cys did not yield a net benefit. However, when considering those who developed RKFD (grey dash line), using FGF-23 in addition to eGFRcr-cys would increase the benefit of using eGFRcr-cys on the basis of subjects' baseline features. The net benefit was calculated as ((proportion of true positives) – (proportion of false positives)*pt)/(1-pt), where pt is the threshold probability (0.18).

Abbreviations: DCA, decision curve analysis; FGF-23, fibroblast growth factor-23; RKFD, rapid kidney function decline

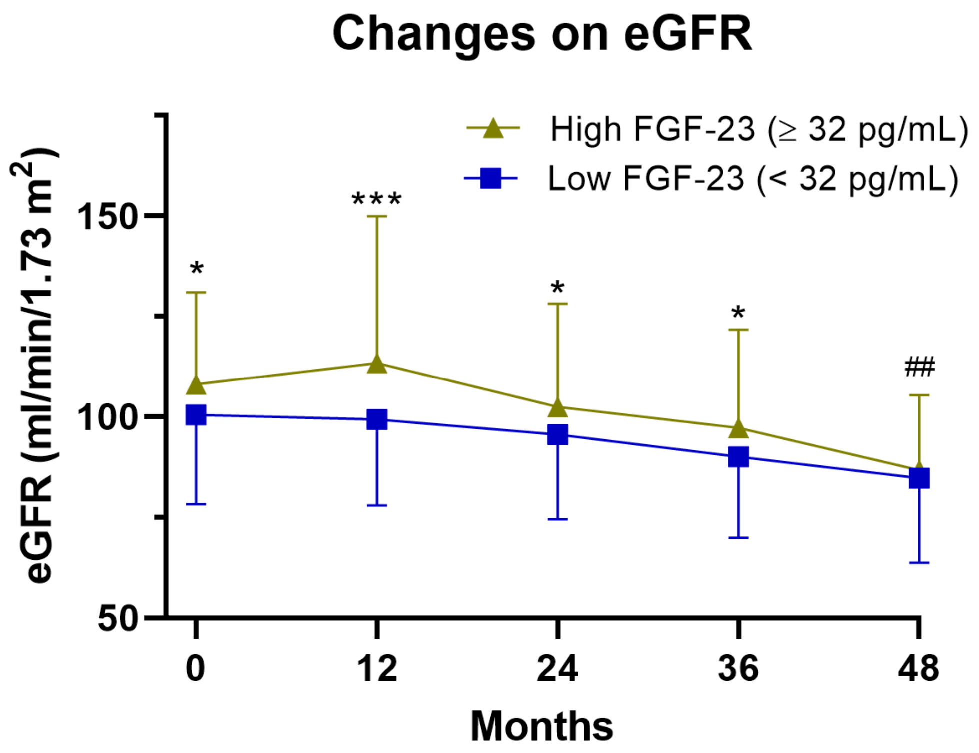

Supplemental Figure S2. Changes in eGFR according to the serum levels of FGF-23 during the study.

Abbreviations: eGFR, estimated glomerular filtration rate; FGF-23, fibroblast growth factor-23; RKFD, rapid kidney function decline; UACR, urine albumin-to-creatinine ratio

P value < 0.01 when comparing the eGFR value of baseline to the end of the follow-up period.

* P value < 0.05 between high and low FGF-23

*** P value < 0.001 between high and low FGF-23

Supplemental Table S1. Social psychology variables of the study population.

	Total (n=220)	RKFD (n=110)	No RKFD (n=110)	p-value
Education level				0.48
Illiterate, n	20 (9.2%)	10 (9.2%)	10 (9.3%)	
Elementary school, n	51 (23.5%)	31 (28.4%)	20 (18.5%)	
Junior high school, n	42 (19.4%)	22 (20.2%)	20 (18.5%)	
Senior high school, n	71 (32.7%)	31 (28.4%)	40 (37.0%)	
Undergraduate, n	30 (13.8%)	13 (11.9%)	17 (15.7%)	
Postgraduate, n	3 (1.4%)	2 (1.8%)	1 (0.9%)	
Substance habituation				
Smoking, n	26 (11.9%)	12 (11.1%)	14 (12.7%)	0.71
Betel nut, n	5 (2.3%)	4 (3.7%)	1 (0.9%)	0.17
Alcohol, n	41 (21.6%)	18 (18.8%)	23 (24.5%)	0.34
Dietary habits				0.95
Non-vegetarian, n	180 (82.6%)	89 (82.4%)	91 (82.7%)	
Vegetarian, n	38 (17.4%)	19 (17.6%)	19 (17.3%)	

Abbreviations: RKFD, rapid kidney function decline

Supplemental Table S2. Hazard ratios estimated by the Cox regression.

	HR (95% CI)	*p*-value	aHR (95% CI)	*p*-value
FGF-23 < 32 pg/mL	reference		reference	
FGF-23 ≥ 32 pg/mL	1.80 (1.22, 2.65)	0.003	2.50 (1.30, 4.79)	0.006

Covariates used in adjusted Cox regression: sex, hypertension, DM, CKD, cardiovascular disease, CVA, HBV, HCV, gout, autoimmune disease, metabolic syndrome, BMI, central obesity, Hgb, total cholesterol, LDL cholesterol, HDL cholesterol, triglyceride, BUN, eGFRcr-cys, uric acid, albumin, GPT, UACR, fasting glucose, HbA1C, insulin, HOMA-IR, total vitamin D, iPTH, P, Ca, OHA use, anti-hypertensives, pain killer use, and dietary habits.

Supplemental Table S3. NRI and IDI analyses for the role of FGF-23 in stratifying individuals into high or low-risk categories (re-classification).

	Model with FGF-23
	Re-classification (%)
Subjects without RKFD	
increased probabilities	30.77
decreased probabilities	69.23
Subjects with RKFD	
increased probabilities	50.00
decreased probabilities	50.00
NRI: 0.384 (Standard error: 0.172), p-value: 0.026	
IDI: 0.073 (Standard error: 0.017), p-value: <0.0001	

Abbreviations: IDI, integrated discrimination improvement; NRI, net reclassification improvement.

Supplemental Table S4. Subgroup analysis of RKFD compared with low (< 32 pg/mL) and high levels (≥ 32 pg/mL) of FGF-23.

	HR (95%CI)	p-value	Wald test
Age, years			0.020
≤60	2.14 (1.27-3.59)	0.004	
>60	1.47 (0.79-2.71)	0.221	
Gender			0.029
Female	1.78 (1.14-2.77)	0.011	
Male	1.94 (0.84-4.50)	0.121	
Hypertension			0.004
no	1.88 (1.18-2.98)	0.007	
yes	1.84 (0.88-3.87)	0.105	
Diabetes			0.004
no	1.73 (1.13-2.65)	0.011	
yes	2.09 (0.79-5.57)	0.139	
Metabolic syndrome			<0.001
no	2.22 (1.37-3.61)	0.001	
yes	1.29 (0.66-2.51)	0.461	
Cardiovascular disease			0.009
no	1.63 (1.08-2.47)	0.020	
yes	4.69 (1.30-16.90)	0.018	
Gout			0.021
no	1.72 (1.15-2.58)	0.008	
yes	3.79 (0.42-33.99)	0.234	

Abbreviation: HR, hazard ratio

Supplemental Table S5. Sensitivity analysis for risk estimation of FGF-23 ≥ 32 pg/mL.

	Unadjusted		Adjusted	
	HR (95%CI)	p-value	HR (95%CI)	p-value
Different PS modeling				
With IPTW (n= 220)	1.42 (0.92-2.19)	0.110	2.20 (1.33-3.64)	0.002
With stratification (n= 220)	1.96 (1.27-3.03)	0.002	3.47 (1.93-6.24)	<0.001
With overlap weighting (n= 220)	1.87 (1.24-2.82)	0.003	3.69 (2.08-6.53)	<0.001
1:1 PSM (n= 96)	1.74 (1.05-2.87)	0.030	7.82 (2.66-22.98)	<0.001

Abbreviations: HR, hazard ratio; IPTW, inverse probability of treatment weighting; PS, propensity score; PSM propensity score matching
